# Potential of Finger Millet Indigenous Rhizobacterium *Pseudomonas* sp. MSSRFD41 in Blast Disease Management—Growth Promotion and Compatibility With the Resident Rhizomicrobiome

**DOI:** 10.3389/fmicb.2018.01029

**Published:** 2018-05-23

**Authors:** Jegan Sekar, Kathiravan Raju, Purushothaman Duraisamy, Prabavathy Ramalingam Vaiyapuri

**Affiliations:** Microbiology Lab, M.S. Swaminathan Research Foundation, Chennai, India

**Keywords:** pseudomonads, bioinoculants, indigenous, compatibility, 2, 4-DAPG, finger millet, formulation, *P. grisea*

## Abstract

Finger millet [*Eleusine coracona* (L). Gaertner] “Ragi” is a nutri-cereal with potential health benefits, and is utilized solely for human consumption in semi-arid regions of Asia and Africa. It is highly vulnerable to blast disease caused by *Pyricularia grisea*, resulting in 50–100% yield loss. Chemical fungicides are used for the management of blast disease, but with great safety concern. Alternatively, bioinoculants are widely used in promoting seedling efficiency, plant biomass, and disease control. Little is known about the impact of introduced indigenous beneficial rhizobacteria on the rhizosphere microbiota and growth promotion in finger millet. Strain MSSRFD41 exhibited a 22.35 mm zone of inhibition against *P. grisea*, produces antifungal metabolites, siderophores, hydrolytic enzymes, and IAA, and solubilizes phosphate. Environmental SEM analysis indicated the potential of MSSRFD41 to inhibit the growth of *P. grisea* by affecting cellular functions, which caused deformation in fungal hyphae. Bioprimed finger millet seeds exhibited significantly higher levels of germination, seedling vigor index, and enhanced shoot and root length compared to control seeds. Cross streaking and RAPD analysis showed that MSSRFD41 is compatible with different groups of rhizobacteria and survived in the rhizosphere. In addition, PLFA analysis revealed no significant difference in microbial biomass between the treated and control rhizosphere samples. Field trials showed that MSSRFD41 treatment significantly reduced blast infestation and enhanced plant growth compared to other treatments. A liquid formulated MSSRFD41 product maintained shelf life at an average of 10^8^ CFU ml^−1^ over 150 days of storage at 25°C. Overall, results from this study demonstrated that *Pseudomonas* sp. MSSRFD41, an indigenous rhizobacterial strain, is an alternative, effective, and sustainable resource for the management of *P. grisea* infestation and growth promotion of finger millet.

## Introduction

The problems associated with indiscriminate use of chemical pesticides in agriculture have led to increasing interest in the use of native and non-native beneficial microorganisms to improve plant health and to increase crop productivity while ensuring food safety and environmental protection (Verma et al., 2013; Santhanam et al., 2015; Souza et al., 2015; Sharma et al., 2017; Schütz et al., 2018). The use of bioinoculants and the exploitation of novel beneficial plant microbes offer promising, sustainable and eco-friendly strategies in conventional and organic agriculture systems worldwide (Negi et al., 2015; Gopalakrishnan et al., 2016; Zhou et al., 2016). Reports have shown that both conventional and organic growers indicate an interest in using microbial inoculants. Approximately 43 million hectares of land have been devoted to organic agriculture using inputs like plant growth promoting bacteria, biocontrol agents, and arbuscular mycorrhizal fungi (AMF) across the globe, and these practices have had positive impacts on health and the quality of food, the environment, and soil (Lernoud and Willer, 2016). The growing demand for bioinoculants is reflected by the fact that in 2016, bioproducts accounted for $1.50 billion and are expected to increase at a compound annual growth rate (CAGR) of 14.1% to reach $3.79 billion by 2023 (Souza et al., 2015; Sekar et al., 2016; Lori et al., 2017; Stratistics Market Research Consulting, 2017).

Among the plant-associated microbes, pseudomonads are dominant bacteria known to protect plants against several phytopathogens (Radjacommare et al., 2005; Waghunde et al., 2013; Yin et al., 2013; Sekar and Prabavathy, 2014; Wang et al., 2015), and to promote plant growth under several abiotic stress conditions (Wang et al., 2015; Jegan et al., 2016). The biocontrol ability of pseudomonads is directly correlated with production of various antibiotics such as 2,4-diacetylphloroglucinol (2,4-DAPG), phenazines, pyrrolnitrin (PRN), pyoluteorin (PLT), hydrogen cyanide (HCN), and lytic enzymes (Radjacommare et al., 2005; Sekar and Prabavathy, 2014; Wang et al., 2015; Ganga et al., 2016). Among these, 2,4-diacetylphloroglucinol (DAPG) exhibits a broad range of antagonistic activity against phytopathogens, and can induce host plant systemic resistance (Weller et al., 2012; Jegan et al., 2016; Viswanath et al., 2016; Yan et al., 2017). Numerous studies on the biocontrol of phytopathogens and plant growth promotion by pseudomonads are available (Yin et al., 2013; Gopalakrishnan et al., 2016; Zhou et al., 2016; Yan et al., 2017), but very little research has been carried out on the control of *P. grisea* by pseudomonads in a highly nutritive crop like millet (Radjacommare et al., 2005; Senthil et al., 2012; Waghunde et al., 2013; Negi et al., 2015).

Nutri-cereals like millets have increasingly gained attention across the world for its health benefits. Finger millet [*Eleusine coracona* (L). Gaertner], also known as “Ragi,” is one of the minor millet cereals which is solely utilized for human consumption in the semi-arid tropics of Asia and Africa (Waghunde et al., 2013; Jegan, 2015; Negi et al., 2015; Kumar et al., 2016; Gupta et al., 2017). This “nutritious millet” offers several health benefits, as it is rich in calcium (0.38%), dietary fiber (18%), and phenolic compounds (0.3–3%). It is also recognized for its beneficial health effects including antidiabetic, antitumorigenic, and atherosclerogenic effects, and antioxidant and antimicrobial properties (Kumar et al., 2016). India is the major producer of finger millet in Asia and it is the staple food for millions of people in the states of Karnataka, Tamil Nadu, Andhra Pradesh, Orissa, Maharashtra, and Bihar, with annual production of 2.2 million tons over an area of 1.6 million ha (Jegan, 2015; Kumar et al., 2016; Gupta et al., 2017).

Although finger millet is considered a hardy crop, it is affected by more than 20 diseases, of which blast disease caused by *Pyricularia grisea* is the most devastating (Prajapati et al., 2013; Waghunde et al., 2013; Magar et al., 2015; Cruz and Valent, 2017). The pathogen infects different parts and stages of the plants from seedling to grain formation, causing diamond-shaped lesions and premature drying of young leaves, affecting the panicle and causing neck and/or finger blast leading to yield loss up to 100% that result in economic loss to farmers and ultimately food crisis (Senthil et al., 2012; Prajapati et al., 2013; Negi et al., 2015). Control of blast disease is a serious and challenging issue relying heavily on chemical pesticides like organophosphorus fungicides which have been reported to be highly effective (Kumar and Kumar, 2011; Magar et al., 2015). However, extended use of chemical pesticides has resulted in the development of pesticide-resistant fungal pathogens, with negative effects on the ecosystem, soil fertility, and water quality, leading to serious health problems including birth defects (Hawkins et al., 2014; Hollomon, 2016). Hence globally, there is a huge demand for pesticide-free food which is safe and nutritious.

Bioinoculants, an alternative to synthetic chemical pesticides, offer multiple beneficial traits; they can ensure the production of quality grains, protect plants against biotic and abiotic stresses; enhance soil fertility, and are sustainable and environmental safe. The development and application of indigenous bioinoculants products has gained momentum among researchers, because they can play a vital role in plant growth promotion and crop protection in sustainable farming systems, and also for their economic value (Schreiter et al., 2014; Santhanam et al., 2015; Sekar et al., 2016; Cai et al., 2017). However, the performance of bioinoculants in the field depends highly on their survival and ability to express key traits in the soil without adversely impacting the native soil microbial community (Gupta et al., 2015; Thomas and Sekhar, 2016; Sharma et al., 2017). Many studies have reported a gradual reduction of introduced bioinoculants in the soil over the period of plant growth, but fewer have addressed the effects of the bioinoculants on the rhizosphere bacterial community (Chowdhury et al., 2013; Yin et al., 2013; Kröber et al., 2014; Schreiter et al., 2014; Thomas and Sekhar, 2016; Sharma et al., 2017). In addition, many bioinoculants have shown promising biocontrol and plant growth promotion under *in vitro* conditions, but exhibit variable performance in greenhouse and field trials (Bulgarelli et al., 2013; Mahmood et al., 2016; Gouda et al., 2018). Hence, identifying suitable crop specific bioinoculants is critical for growth promotion and disease suppression under variable ecological conditions. Therefore, this study attempted to investigate the efficiency of indigenous rhizosphere *Pseudomonas* sp. MSSRFD41 for plant growth promotion and protection against the *P. grisea* blast pathogen under *in vitro* and *in vivo* conditions. *Pseudomonas* sp. MSSRFD41, isolated from the rhizosphere of finger millet in India, is a novel 2,4-DAPG-producing strain with potential biocontrol and plant growth promoting traits (Sekar and Prabavathy, 2014). The present investigation aims (i) to assess the potential traits of MSSRFD41 involved in the inhibition of *P. grisea, in vitro*; (ii) to determine the survival of MSSRFD41 in the rhizosphere and its impact on the rhizosphere microbial community; and (iii) to determine the efficacy of MSSRFD41 in blast disease control and growth promotion in finger millet under field conditions.

## Materials and methods

### Source and growth conditions of strain MSSRFD41 and *P. grisea*

*Pseudomonas* sp. MSSRFD41 was isolated from the rhizosphere of finger millet cultivated in the Dharmapuri district of Tamil Nadu, India (Sekar and Prabavathy, 2014). The active culture was maintained in King's B agar (KBA) medium (King et al., 1954) at 28°C and stored at −80°C in phosphate-buffered 20% (v/v) glycerol. *P. grisea* TN508 was obtained from Tamil Nadu Agricultural University culture collection center—Coimbatore and maintained in Oat meal agar and sterile finger millet seeds.

### *In vitro* screening for antifungal activity of MSSRFD41

The antagonistic activity of MSSRFD41 was assayed against the blast pathogen, *P. grisea* TN508 by the dual plate method in KBA agar medium. A 6 mm diameter plug of actively growing 10 days old fungal culture was inoculated in the middle of the agar plates and incubated at 28°C for 48 h. Later, a loopful of an actively growing culture of the *Pseudomonas* was inoculated 3.5 cm away from the fungal disc on two sides. Plates without MSSRFD41 served as control. The plates were incubated at 28°C for 10 days and examined for inhibition of fungal growth by MSSRFD41.

### Profiling of crude metabolite by GC-MS

Crude metabolites were extracted from 100 ml broth of MSSRFD41 grown in King's B medium at 28°C for 48 h. The supernatant was collected by centrifuging at 9,300 g for 10 min and the pellet was discarded. The supernatant was acidified with concentrated HCl to pH 2.0 and then extracted twice with an equal volume of ethyl acetate. The extract was pooled and concentrated using a rotary evaporator at 35°C and then stored at −20°C for further use. The concentrated crude metabolites obtained from the culture broth were dissolved in 1 ml methanol: chloroform mixture (1:1) and analyzed by GC-MS (MassHunter GC/MS, Agilent Technologies, US). The compounds were identified by comparison of mass spectra with the National Institute of Standards and Technology (NIST database) library and by direct comparison with published data (Dheepa et al., 2016).

### Environmental scanning electron microscope analysis of *P. grisea* mycelium

Morphological changes of the hyphae of *P. grisea* in control cultures and cultures co-inoculated with MSSRFD41 were determined through Environmental Scanning Electron Microscopy (ESEM). Mycelial bits were cut from the actively growing edge of the fungal cultures and directly subjected to ESEM analysis. Images of samples were taken by FEI Quanta 200—High-Resolution Scanning Electron Microscope at a voltage of 8 kV and a pressure from 500 to 600 Pa.

### Determination of hydrolytic enzyme activity

Strain MSSRFD41 were tested for the production of chitinase as described by Kole and Altosaar (1985) in Dworkin-Foster (DF) salts minimal medium containing 2.5% (w/v) colloidal chitin. Cellulase activity was determined in carboxymethyl cellulose (CMC) agar containing 5% (w/v) CMC (Sigma Aldrich, USA) (Ariffin et al., 2008). Proteolytic activity was assessed using skimmed milk agar (Hi Media, India) (Wikström, 1983).

A overnight grown culture of MSSRFD41 was inoculated into media containing peptone 10 g l^−1^, NaCl 5 g l^−1^, CaCl_2_ 2H_2_O 0.1 g l^−1^, agar 18 g l^−1^, and 1% of sterilized Tween 80 (Hi Media, India) for detection of esterase activity or Tween 20 (Hi Media, India) for lipolytic activity as described by Sierra (1957). After incubation at 28°C for 48 h, a clear halo zone around the colony was considered as positive.

Amylase activity of MSSRFD41 was determined by inoculating in starch agar (Hi Media, India) plates containing starch as the only carbon source. After incubation at 28°C for 48 h, plates were stained with Gram's iodine solution and the formation of a clear halo zone in the starch agar around the colony indicated amylase production (Cappuccino and Sherman, 1992).

Strain MSSRFD41 was assessed for inorganic phosphate solubilization by inoculating a freshly grown culture in the National Botanical Research Institute's phosphate (NBRIP) agar medium contained glucose, 10 g l^−1^, Ca_3_(PO_4_)_2_, 5 g l^−1^, MgCl_2_·6H_2_O, 5 g l^−1^, MgSO_4_·7H_2_O, 0.25 g l^−1^, KCl, 0.2 g l^−1^, (NH_4_)_2_SO_4_, 0.1 g l^−1^, and agar 18 g l^−1^ (Nautiyal, 1999). The plates were incubated at 28°C for 5 days and the diameter of a clear halo zone around the bacterial colony indicating solubilization of mineral phosphate was measured.

### Siderophores production

Strain MSSRFD41 was grown overnight at 28°C in King's B broth and spotted on Blue Agar Chromeazurol “S” plates and incubated at 28°C. After incubation, production of siderophores was detected by the appearance of orange-halo zones against a blue background (Louden et al., 2011).

### Quantification of indole-3-acetic acid

An overnight culture of strain MSSRFD41 grown on Luria-Bertani (LB) medium was inoculated into tubes containing 5 ml LB broth supplemented with 100 μg ml^−1^ L-tryptophan and incubated at 28°C. Samples were collected after 72 h of incubation and the bacterial cells were removed by centrifugation at 6,500 g for 10 min. A 1 ml aliquot of the supernatant was mixed vigorously with 4 ml of Salkowski's reagent and incubated in the dark for 20 min at room temperature. IAA production was observed as the development of a pink-red color and absorbance was measured at 535 nm using a spectrophotometer. The concentration of IAA was quantified by comparison with a standard curve prepared with 5–100 μg ml^−1^of pure IAA (Sigma Chemicals, India) (Patten and Glick, 2002).

### Ammonia production

Ammonia production was assessed qualitatively by inoculating 10 ml of peptone broth with strain MSSRFD41 and incubating the tubes at 28°C for 48 h. After incubation, drops of Nessler's reagent (Hi Media, India) were added and development of a yellowish brown color indicated the production of ammonia (Cappuccino and Sherman, 1992).

### Impact of MSSRFD41 on soil and rhizobacterial isolates

The compatibility and impact of strain MSSRFD41 on 129 isolates from indigenous and non-indigenous rhizosphere and soil sources were assessed by cross-streaking. The isolates were obtained from the M.S. Swaminathan Research Foundation (MSSRF) culture collection and are described in Table S1. A fresh culture of MSSRFD41 grown overnight at 28°C in King's B broth was streaked across the center of a KBA plate and the test isolates were streaked at right angles to MSSRFD41. The plates were incubated at 28°C for 72 h and observed for growth inhibition.

### *In vitro* and *in vivo* plant growth stimulation by MSSRFD41

Cultures of strain MSSRFD41 were grown overnight in 50 ml KB broth at 28°C on a rotary shaker at 180 rpm. The bacterial culture was pelleted by centrifugation at 6,500 × g for 10 min and the supernatant was discarded. Bacterial pellets were washed twice with 5 ml of sterile 0.03 M MgSO_4_, and the final suspension was adjusted spectrophotometrically to an OD_600_ of 0.5 with 0.03 M MgSO_4_, corresponding to a cell density of 10^8^ cells ml^−1^.

To determine colonization efficiency, finger millet seeds were surface sterilized and inoculated with a bacterial suspension following the method described by Patten and Glick (2002). Approximately 50 g seeds were surface sterilized by washing in 10 ml of 70% ethanol for 1 min and residues was removed by washing the seeds five times with sterile distilled water. The sterilized millet seeds were incubated with 5 ml of bacterial suspension (OD_600_ of 0.5) at room temperature for 4 h to allow the bacteria to bind to the seed, and control seeds were incubated in sterile 0.03 M MgSO_4_ under the same conditions. The treated and control seeds were spread on KBA medium and incubated overnight at room temperature to check for contamination.

The impact of seed priming with strain MSSRFD41 was assessed with ten seeds per treatment that were transferred aseptically to presoaked sterile germination paper, aseptically rolled, and placed inside a test tube containing sterilized half-strength Hoagland's solution (ISTA, 1993). Tubes were incubated in a growth chamber with an 8 h of darkness and 16 h of light and 65% humidity at 25°C. On the 4, 6, and 8th day of incubation, 3 tubes per treatment were removed from the growth chamber and biometric parameters *viz*., seed germination, root and shoot length, and fresh and dry biomass were measured. The seed vigor index between treatments was calculated by using the formula: Vigor index = (Mean root length + Mean shoot length) X Germination (%) (Abdul-Baki and Anderson, 1973).

For pot experiments, millet seeds were surface sterilized, as described above and germinated in a seedling tray filled with vermiculite. Fifteen days after sowing, seedlings were dipped in an inoculum of MSSRFD41 (10^8^ CFU ml^−1^) and control seedlings were dipped into water. After 1 h of incubation, two seedlings were transplanted from the treatment to the respective pots (17 × 17 × 16 cm) filled with 2.5 kg of agricultural soil. The treatments were arranged in a completely randomized block design with four pots of each replication and three replications per treatment. Pots were maintained under greenhouse conditions at 35 ± 2°C with a photoperiod of 12/ 12 h (light/ dark) and watered periodically. Plant growth response biometric parameters including shoot and root length, straw fresh and dry weight, the fresh and dry weight of root were measured at 20, 60, and 100 days after transplantation (DAT).

### Rhizosphere colonization and population dynamics of strain MSSRFD41

Root adherent soils were collected by uprooting the control and MSSRFD41 treated seedlings of 20, 60, and 100 days old. The cultivable rhizobacterial population dynamics were determined using Kings B agar—KBA, and pseudomonads community was assessed using selective media like Kings B agar + sodium lauroyl sarcosine + Trimethoprim (20 mg^−1^)—KBA+SLST (Gould et al., 1985) and Kings B agar + Cetrimide + nalidixic acid (15 μg ml^−1^)—KBA+CN (Goto and Enomoto, 1970). The rhizosphere soil samples were diluted up to 10^−6^ in 1% (v/v) PBS (phosphate buffered saline—pH 7.2) and plated onto the above media in triplicates. The plates were incubated at 28°C for 3 to 4 days and log CFU g^−1^ fresh weight of rhizosphere soils were determined.

To monitor populations of strain MSSRFD41 over time, bacterial colonies were picked from the dilution plates of selective media from both the control and treated samples and streaked on fresh KBA medium. DNA was extracted from isolated rhizobacteria and DNA fingerprinting analysis was carried out with BOX primer 5′-ACG GCA AGG CGA CGC TGA CG-3′ and compared to reference DNA from strain MSSRFD41 as described by Jegan (2015). The BOX-PCR genetic patterns were visualized by UV illumination at 365 nm and documented using a Bio-Rad Gel Doc system (Bio-Rad, USA). Normalization, recognition, and assignment of bands on the gel were performed with the GelJ analysis DNA fingerprint tool by the Dice coefficient (Heras et al., 2015). The unweighted pair group with mathematical average (UPGMA) algorithm was performed for cluster analysis with similarity matrices for generation of a dendrogram. Rhizobacterial isolates showed similar fingerprinting pattern to reference DNA were identified using 16S rRNA sequencing as described by Sekar and Prabavathy (2014).

### Phospholipid fatty acid (PLFA) analysis

Rhizosphere soil samples were collected from 100-day-old control and MSSRFD41-treated plants, were pooled according to treatment, and outsourced to the Royal Research Laboratories (Secunderabad, India) for PLFA analysis and to calculate the biomass (nmol g^−1^ of dry rhizosphere soil) for each microbial type present. Samples were extracted as described by (Buyer and Sasser, 2012) and analyzed by gas chromatography and the MIDI Sherlock Software v.6.2B PLFA Package (Agilent 6890N Series). Sherlock PLFA Tools software was used to calculate the biomass, mole percent, and key PLFA ratios nmol g^−1^ of rhizosphere soil dry weight.

### Field trial

A field trial was conducted in a farmer's field located in Ramiyanahalli, Pappireddipatti taluk Dharmapuri district, Tamil Nadu (12°02′16.7″N 78°22′21.5″E). The trial consisted of five treatments T1–Control; T2–chemical (Carbendazim−2 g^−1^); T3–C-PF (commercial Ecomonas *P. fluorescens*−10 g^−1^); T4–Bio + 50% chemical (*Pseudomonas* sp. MSSRFD41–5 ml^−1^ + Carbendazim−1 g^−1^); and T5–MSSRFD41 (5 ml^−1^). Farmyard manure at the rate of 7.5 t per ha were applied to the field and plot layouts were prepared in Randomized Block Design (RBD) in triplicates with an individual plot size of 6 × 4 m (Figure S1). One kg of seeds for each treatment were treated with the above as described dose, dried in the shade for 3 to 4 h, and sown in the respective seed beds. After 25 days, seedlings were uprooted, dipped into the respective treatments, incubated for 1−2 h and planted, two seedlings per hill, at a depth of 4−5 cm with a distance of 10 cm between seedlings in the respective treatments plots. Foliar sprays were given separately to the plots according to the treatment after 30 and 60th DAT. The percentage of natural disease incidence was assessed at 90th day by monitoring blast infestation in plants using the following formula:

$$\begin{array}{l} \text{Percent disease incidence }=\;\frac{\text{Number of infected plants}}{\text{Total number of plants}}\times 100 \end{array}$$

After 90 days randomly 20 plant samples were harvested from each plot and the root, shoot length, and 1,000 seed weight were measured.

### Preparation of liquid-based formulation of MSSRFD41

An overnight culture of MSSRFD41 grown in 100 ml KB broth with 5.08 log CFU ml^−1^ was pelleted and suspended in 1 liter of sterilized KB broth with 5% polyvinylpyrrolidone (PVP), 3% glycerol and 1.5% polyethylene glycol 6000 (PEG). The formulated product was incubated at 4, 8, 27, and 37°C in static conditions in triplicate. The viable population in the formulated product was determined at 3, 7, 15, 30, 60, 90, 120, and 150 days of storage by serial dilution and plating on nutrient agar medium (Goljanian-Tabrizi et al., 2016).

### Data analysis

Data were analyzed using SPSS (Version 22) and GraphPad Prism 6. Significant differences among treatments were determined by applying a one-way ANOVA, and Tukey's Honestly Significant Difference (Tukey's HSD) *post-hoc* tests were used for mean separation when ANOVA results were significant (*P* < 0.05).

## Results

### Antifungal effect of MSSRFD41

**Figure 1:**
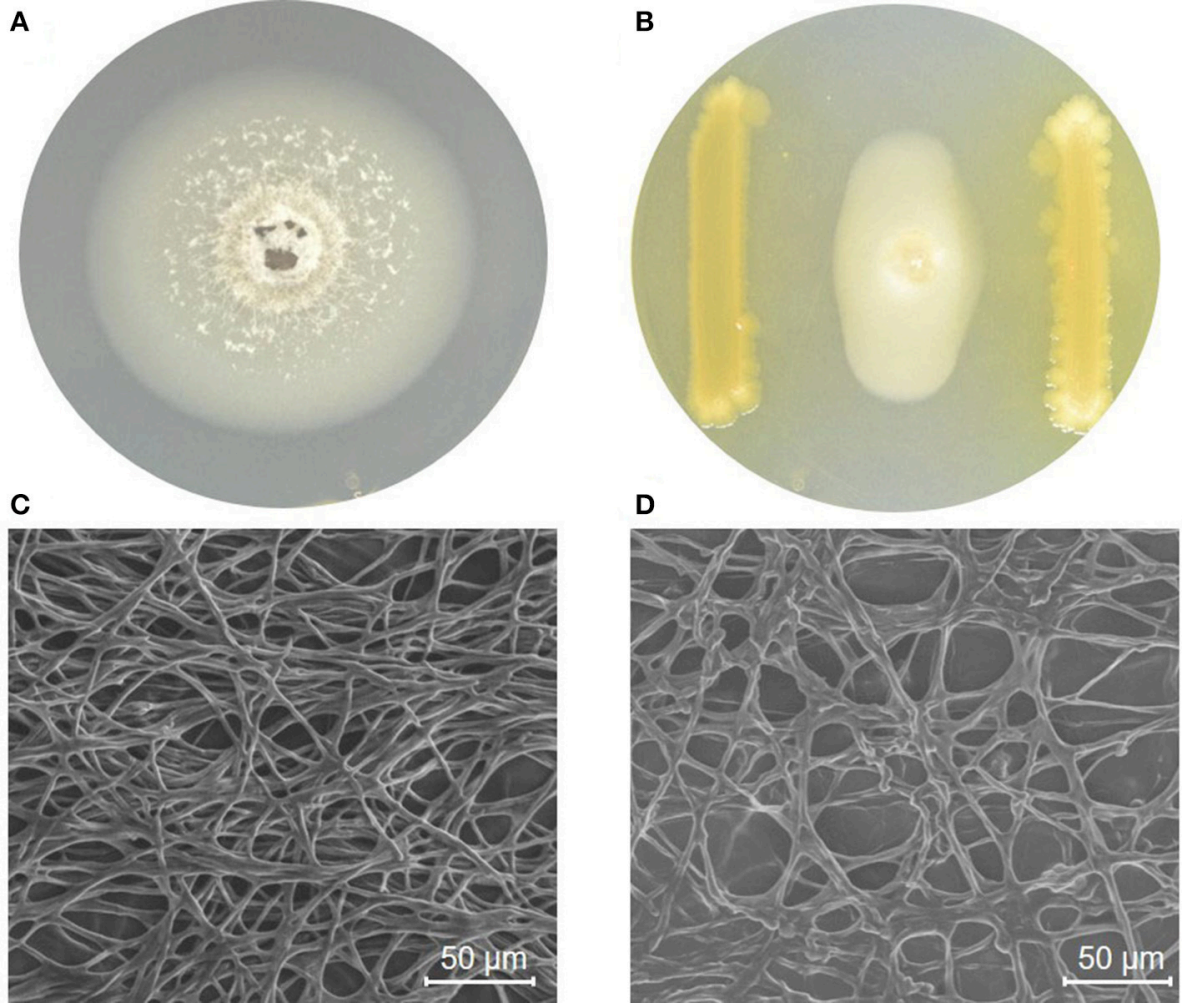
MSSRFD41 inhibiting mycelial growth of *P. grisea* in a dual plate culture. **(A)** *P. grisea* control; **(B)** *P. grisea* with MSSRFD41. Scanning electron microscopic studies on the impact of MSSRFD41 on hyphae of *P. grisea*, **(C)** Control hyphae without MSSRFD41; **(D)** Antagonized hyphae with MSSRFD41.

*P. grisea* TN508 inoculated in control KBA plates showed regular growth without any inhibition of mycelial growth (Figure 1A). In dual culture plates, strain MSSRFD41 showed significant inhibition of *P. grisea* TN508 mycelial growth, producing a 22.35 mm zone of inhibition (Figure 1B). GC-MS of crude metabolite revealed the presence of 47 different metabolites which included bioactive compounds in the groups of acids, esters, alcohols, nitrogenous compounds, aldehydes, and volatiles. Based on the retention time and comparison with NIST database presence of potential antifungal compounds including 2,4-DAPG derivatives, octasiloxane, pyrrolo, 2,5-piperazinedione, 1,2-benzenedicarboxylic acid, hexadecanoic acid, octadecenoic acid, pyran, propenoic acid, and dasycarpidan was detected (Table 1). SEM analysis was employed to examine the structural changes of *P. grisea* in control and dual culture plates with strain MSSRFD41. Mycelia obtained from the edges of the fungus grown in control plates showed hyphae with typical net structure with smooth surfaces lacking visible damage (Figure 1C). Co-culturing with strain MSSRFD41 resulted in the presence of damaged hyphae showing loss of smoothness, aberrant shape, and forming unusual bulges in the hyphal network (Figure 1D).

**Table 1:** Antimicrobial compounds identified from *Pseudomonas* sp. MSSRFD41 strain through GC/MS.

**S. No**	**Name of the compound**	**Peak Number**	**Peak Area %**	**Retention time**
1.	3,3,3-Trifluoro-2-(2-methoxy-benzoylamino)-2-(4-methyl-thiazol-2-ylamino)-propionic acid methyl ester	8	9.24	4.895
2.	Z-10-Methyl-11-tetradecen-1-ol propionate	13	9.9	5.164
3.	9-Octadecenoic acid, (2-phenyl-1,3-dioxolan-4-yl)methyl ester, trans	16	16.55	5.39
4.	2-Propenoic acid, 3-phenyl-	21	30.34	5.755
5.	1-Heptatriacotanol	27	5.45	6.269
6.	Phenol, 2,5-bis(1,1-dimethylethyl)-	28	54.03	6.346
7.	Phen-1,4-diol, 2,3-dimethyl-5-trifluoromethyl-	30	41.1	6.485
8.	2H-Pyran, 2-(7-dodecynyloxy)tetrahydro-	33	10.52	6.793
9.	2,5-Piperazinedione, 3-methyl-6-(1-methylethyl)-	37	13.22	7.549
10.	(3S,6S)-3-Butyl-6-methylpiperazine-2,5-dione	40	29.33	8.508
11.	1,2-Benzenedicarboxylic acid, bis(2-methylpropyl) ester	49	5.43	10.129
12.	n-Hexadecanoic acid	54	42.46	11.376
13.	7-Methyl-Z-tetradecen-1-ol acetate	57	26.95	12.364
14.	Piperidine-3-carboxylic acid, 1-(5,8a-dimethyl-2-oxo-2,3,3a,7,8,8a,9,9a-octahydronaphtho[2,3-b]furan-3-ylmethyl)-, ethyl ester	61	43.21	14.234
15.	tert-Hexadecanethiol	65	24.17	14.878
16.	Pyrrolo[1,2-a]pyrazine-1,4-dione, hexahydro-3-(2-methylpropyl)	69	44.19	17.706
17.	Dasycarpidan-1-methanol, acetate (ester)	70	34.02	17.99

### Physiological traits detected *in vitro*

MSSRFD41 formed clear zones around colonies grown in the presence of chitinase (8 mm), protease (25 mm), cellulase (11 mm), esterase (13 mm), lipase (10 mm), and amylase (21 mm) (Table 2). Phosphate solubilization was observed in NBRIP medium as a 7.08 mm clearance zone around the colony. Siderophore production was detected on the basis of a 12.8 mm zone of color change from blue to orange in CAS medium of 12.8 mm. In the presence of tryptophan, strain MSSRFD41 produced IAA at a concentration of 29 μg ml^−1^ after 72 h, and 29 μg ml^−1^ of ammonia was detected from peptone broth. Dual cultures of different rhizobacterial isolates and strain MSSRFD41 revealed no growth inhibition (Figure S2), indicating that the strain was compatible with groups of indigenous and non-indigenous rhizobacteria. This pilot scale assay revealed strain MSSRFD41 is compatible in the rhizosphere without impairment to other rhizobacterial isolates.

**Table 2:** Biocontrol and plant growth promoting traits of *Pseudomonas* sp. MSSRFD41.

**S. No**	**Traits**	**Zone of Inhibition (or) Production**
1.	*P. grisea*	22.35 ± 0.88mm
2.	Chitinase	8.02 ± 0.53mm
3.	Protease	24.98 ± 0.78mm
4.	Cellulase	10.96 ± 0.65mm
5.	Esterase	13.12 ± 0.48mm
6.	Lipase	10.28 ± 0.68mm
7.	Amylase	21.34 ± 0.39mm
8.	Phosphate solubilization	7.04 ± 0.48mm
9.	Siderophore production	12.8 ± 0.86mm
10.	IAA production	29.09 ± 0.30μg/ml
11.	Ammonia production	13.20 ± 0.57μg/ml

*Each value represents the mean ± SD (n = 5)*.

### Effect of MSSRFD41 biopriming on finger millet germination and vigor

Priming of finger millet seeds with a suspension of MSSRFD41 had a significant effect on the germination percentage, plant biomass and vigor index compared to the control at different time intervals. Treated millet seeds showed significantly increased germination by 7.44% compared to control on the 8th day of incubation, while the vigor index was increased by 21.58%. Priming of millet seeds with MSSRFD41 significantly improved the length of shoots (19.58%) and roots (11.65%) compared to the control (Figure S3). Increased germination rate, plant length and vigor index were observed in the MSSRFD41 primed seedlings (Table 3). At all-time points, the treated seeds performed better than the control, suggesting that priming could enhance the germination, biomass, and vigor of millet seeds.

**Table 3:** Biopriming effect of MSSRFD41 on finger millet seedling growth.

**Days**	**Treatments**	**Germination %**	**Shoot Length (cm)**	**Root length (cm)**	**Plant biomass (g)**	**Vigor Index**
4	Control	82.11 ± 2.1^a^	1.53 ± 0.09^a^	3.47 ± 0.24^a^	0.10 ± 0.001^a^	411.70 ± 3.04^a^
	Treated	88.89 ± 2.8^b^	1.65 ± 0.13^a^	4.56 ± 0.17^b^	0.12 ± 0.001^b^	552.73 ± 3.14^b^
6	Control	87.44 ± 2.3^ab^	2.26 ± 0.49^b^	5.94 ± 0.29^c^	0.14 ± 0.001^c^	718.13 ± 3.01^c^
	Treated	96.77 ± 2.0^c^	2.93 ± 0.69^c^	6.87 ± 0.22^d^	0.16 ± 0.005^d^	949.47 ± 2.87^d^
8	Control	90.07 ± 2.0^b^	3.04 ± 0.75^c^	8.73 ± 0.46^e^	0.17 ± 0.002^d^	1060.67 ± 3.66^e^
	Treated	97.44 ± 2.6^c^	3.70 ± 0.81^d^	9.81 ± 0.18^f^	0.21 ± 0.003^e^	1316.66 ± 2.95^f^

*Each value represents the mean ± SD (n = 30) and within a column different letters were assigned when values were significantly different according to the HSD Tukey test (P < 0.05)*.

### Impact of MSSRFD41 on growth promotion of finger millet in pots

Under greenhouse conditions, seedlings treated with strain MSSRFD41 had increased shoot (61.54 cm) and root length (17.30 cm) that were significantly greater than those of control seedlings, with differences of 13–26% among treatments and days after transplanting (DAT). The maximum straw fresh weight (8.43 g), straw dry weight (3.53 g); root fresh weight (4.01 g) and root dry weight (2.58 g) were observed from samples of 100 DAT. Values for straw fresh weight (6.84 g), dry weight (2.42 g), root fresh weight (3.07 g), and dry weight (1.78 g) were lower for the control seedlings (Figure S5). At 20 DAT, treated and control seedlings did not differ significantly by biometric analysis, but the 60 and 100 day biometric data were significant between the treatments (Table 4).

**Table 4:** Effect of MSSRFD41 priming on growth of finger millet seedling in pots.

**DAT**	**Treatments**	**Shoot length (cm)**	**Root length (cm)**	**Straw fresh weight (g)**	**Straw dry weight (g)**	**Root fresh weight (g)**	**Root dry weight (g)**
20	Control	6.64 ± 0.98^a^	2.99 ± 0.63^a^	0.73 ± 0.16^a^	0.47 ± 0.20^a^	0.17 ± 0.06^a^	0.10 ± 0.02^a^
	Treated	7.94 ± 0.78^a^	3.50 ± 0.43^a^	1.02 ± 0.27^a^	0.76 ± 0.22^a^	0.29 ± 0.11^a^	0.20 ± 0.07^ab^
60	Control	48.27 ± 1.80^b^	8.98 ± 2.7^b^	3.18 ± 0.81^b^	1.51 ± 0.33^b^	1.97 ± 0.43^b^	1.17 ± 0.49^bc^
	Treated	54.60 ± 2.95^c^	12.46 ± 1.53^c^	4.84 ± 0.88^c^	2.77 ± 0.522^c^	2.89 ± 1.13^c^	1.89 ± 0.69^cd^
100	Control	53.70 ± 2.53^c^	13.37 ± 2.49^c^	6.84 ± 1.17^d^	2.42 ± 0.23^c^	3.07 ± 0.23^c^	1.78 ± 0.42^c^
	Treated	61.54 ± 2.98^d^	17.30 ± 2.87^d^	8.43 ± 1.66^e^	3.53 ± 0.41^d^	4.01 ± 0.56^d^	2.58 ± 1.04^d^

*Each value is the mean ± SD (n = 5) and within a column different letters were assigned when differences were significant according to a HSD Tukey test (P < 0.05)*.

### Survival of MSSRFD41 and dynamics of rhizobacterial populations

**Figure 2:**
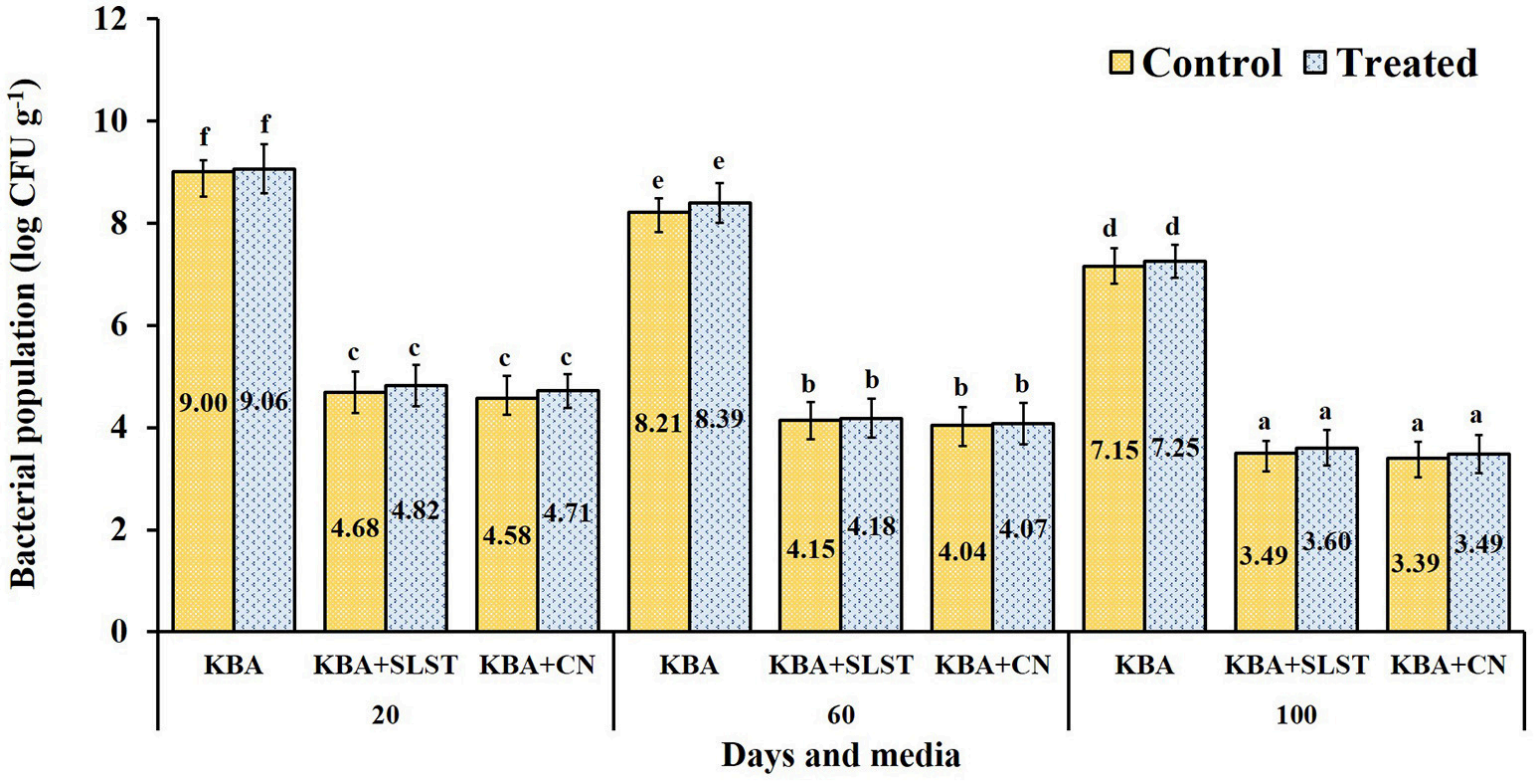
Cultivable rhizobacteria isolated from rhizosphere samples of treated and control finger millet. KBA–Kings B agar; KBA+SLST–Kings B agar + sodium lauroyl sarcosine + Trimethoprim (20 mg^−1^); KBA+CN–Kings B agar + Cetrimide + nalidixic acid (15 μg ml^−1^). Each value represents the means ± *SD* of 5 replicates and within a column, different letters were assigned when significant according to HSD Tukey test (*P* < 0.05).

Root colonization and establishment in the rhizosphere are vital for any potential bioinoculants. Rhizobacterial populations from 20-day roots of treated millet seedlings grown in pots harbored cultivable bacterial population of 9.06 log CFU g^−1^ (KB); and in pseudomonads selective media 4.82 log CFU g^−1^ in KBA+SLT; and 4.71 log CFU g^−1^ in KBA+CN were recorded. In control seedlings, the populations were 9.00 log CFU g^−1^ (KB); 4.68 log CFU g^−1^ (KBA+SLT); 4.58 log CFU g^−1^ (KBA+CN). The 60-day roots collected from treated seedlings harbored 8.39 log CFU g^−1^ (KB); 4.38 log CFU g^−1^ (KBA+SLT); 4.07 log CFU g^−1^ (KBA+CN); in control seedlings the cultural populations of 8.21 log CFU g^−1^ (KB); 4.15 log CFU g^−1^ (KBA+SLT); 4.04 log CFU g^−1^ (KBA+CN) were observed. At 100 days, the rhizobacterial population from the roots decreased slightly in treated and control plants. Overall, among the treatments no significant difference in total rhizobacterial population was detected (*P* = 0.503), but was highly significant in CFU g^−1^ of the samples collected at different days (*P* = < 0.0001) and media (*P* = < 0.0001) (Figure 2). At all-time points and on all the media, greater numbers of CFU were observed on roots from treated than from control plants. These results confirm that the introduced MSSRFD41 had no significant impact on rhizobacterial population dynamics.

### Monitoring of MSSRFD41 in the rhizosphere via BOX-PCR

**Figure 3:**
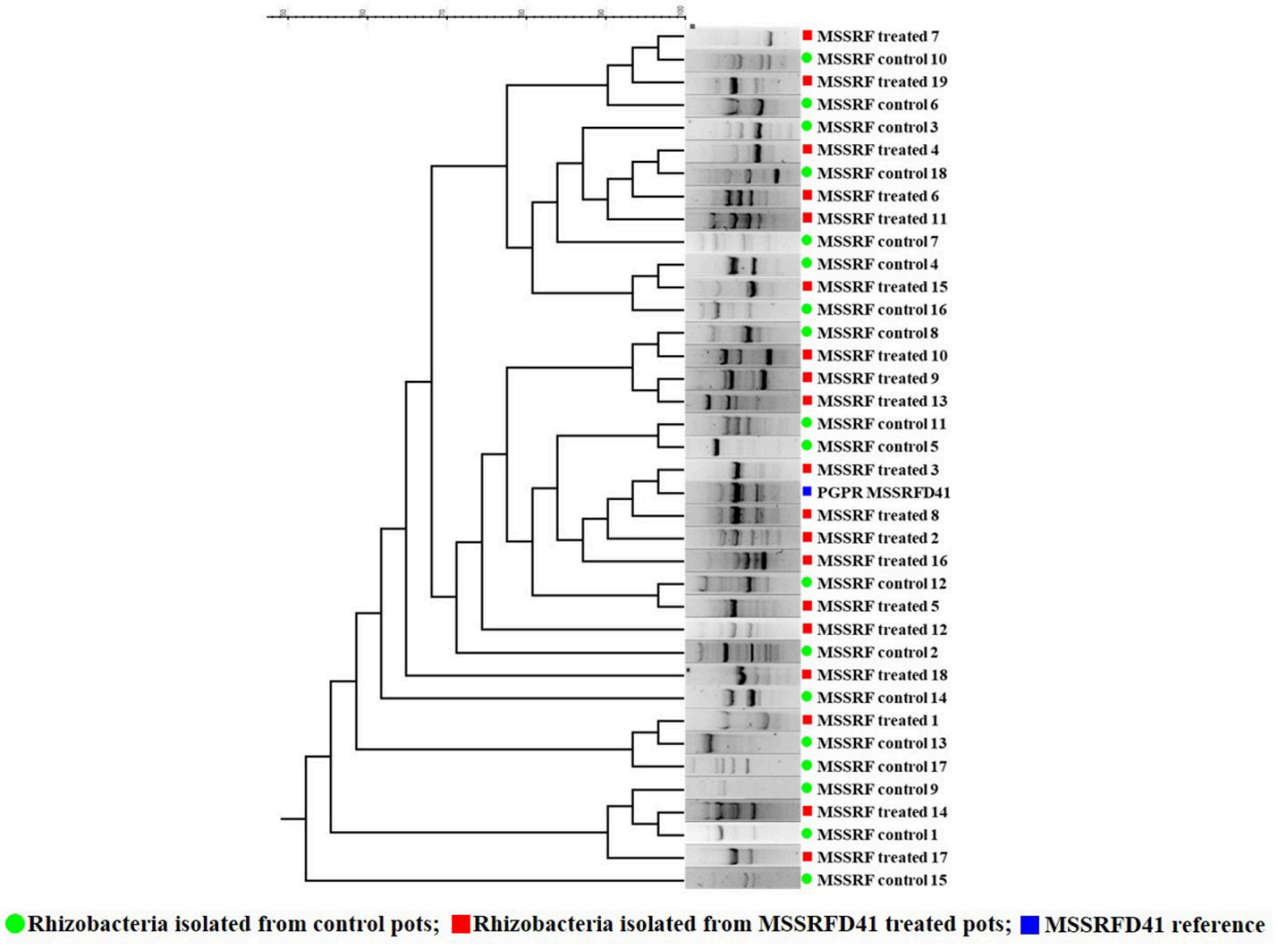
Genomic fingerprinting to evaluate MSSRFD41 colonization and survival.

Based on the rhizobacterial colony morphotypes, total of 37 rhizobacteria were isolated of which 19 isolates from treated and 18 from control samples were recovered from the 100-day plant rhizosphere soil plated on KB, KBA+SLT, and KBA+CN media. BOX-PCR profiles of the amplified products ranged from 200 to 6,000 bp. BOX fingerprinting revealed that isolates 3 and 8 obtained from samples that had been treated with strain MSSRFD41 showed a banding pattern similar to that of MSSRFD41 reference DNA and formed a monophyletic cluster at a similarity coefficient value of 100% (Figure 3). In addition, the diversity among the total rhizobacterial isolates from the treated and control samples were identical at a similarity coefficient value of 80%. This strongly confirms the colonization of MSSRFD41 in the rhizosphere of the plant at harvest without any impact on the existing rhizobacterial community.

16S rRNA gene (~1450 bp) sequencing of the rhizobacterial isolates MSSRF treated 3 (Accession no. MH071151) and MSSRF treated 8 (Accession no. MH071151) showed 100% similarity index to the reference MSSRFD41 (Accession no. MG738708) and *Pseudomonas* sp in the BLAST analysis. Phylogenetic relationship among the isolates showed that rhizobacterial isolates MSSRF treated 3 and 8 forms clade with MSSRFD41, it clearly indicates that both isolates are similar to the reference MSSRFD41 (Figure S4).

### PLFA analysis

PLFA profiles of the rhizosphere samples of 100-day-old plants indicated total PLFA biomass content in control and treated samples, respectively, of 1,451 and 1,459 nmol g^−1^. Measures of the treated and control rhizosphere samples indicated no significant differences in biomass of Gram-positive/Gram-negative bacteria, anaerobes, actinomycetes, fungi, eukaryotes, not assigned, and total PLFA groups (Table 5). These results indicate that strain MSSRFD41 did not inhibit (or) significantly alter the normal flora of rhizosphere microbial community, consistent with the results of the culturable population analysis.

**Table 5:** Soil microbial biomass analysis in treated pots by PLFA.

**PLFAs**	**Biomass content in control samples (nmol g^−1^ soil)**	**Biomass content in treated samples (nmol g^−1^ soil)**
Gram Positive	301.11 ± 2.55	303.66 ± 2.52
Gram Negative	530.72 ± 3.79	535.12 ± 3.06
Anaerobe	9.06 ± 0.87	9.12 ± 0.87
Actinomycetes	17.59 ± 1.48	17.97 ± 1.15
Fungi	7.47 ± 0.45	7.25 ± 0.60
Eukaryote	333.39 ± 3.25	334.30 ± 2.65
Not Assigned	251.71 ± 2.51	251.59 ± 2.01
**Total PLFA**	**1451.05** ± **3.14**	**1459.02** ± **2.15**

*Each value represents the mean ± SD (n = 3) and no significant difference was observed between the treatments*.

### Efficacy of MSSRFD41 in control of blast disease and growth promotion under field conditions

A field trial revealed the potential ability of MSSRFD41 to control the blast disease caused by *P. grisea* as well as to promote growth of finger millet compared to other treatments (Table 6). All the treatments showed a significant difference in the suppression of disease occurrence, but plots treated with strain MSSRFD41 showed lower incidence of 8.39%, three-fold lower than that of the control treatment. In the Bio + 50% chemical treatment plots, 11.42% disease incidence was observed, which was significantly lesser than the chemical (13.26%), C-PF (16.73%), and control (26.87%) treatments. Plant growth assessment showed maximum root length (19.05 cm) and shoot length (64.68 cm) in plants that had received the MSSRFD41 treatment which was significantly greater than values observed for other treatments including the Bio + 50% chemical treatment (root length 18.15 cm and shoot length 63.42 cm). All the four treatments showed significantly higher root and shoot lengths than the control treatment, but between the chemical and C-PF treatments, no significant difference was observed. Among the treatments, MSSRFD41-treated plots had 1,000 seed weight (3.23 g) that was significantly higher than the results from the other treatments. But no critical difference in 1,000 seed weight was detected among chemical, C-PF, and Bio + 50% chemical treatments. Overall the MSSRFD41 treatment evidenced control of blast disease incidence and enhancement of millet growth greater than that of the chemical treatment, which supports the idea that it can be used as a potential bioinoculant for finger millet crop protection and growth promotion.

**Table 6:** Field efficacy of *Pseudomonas* sp. MSSRFD41 in the control of *P. grisea* and growth promotion of finger millet.

**Treatments**	**Percent disease incidence**	**Shoot length (cm)**	**Root length (cm)**	**1000 seed weight (g)**
Control	26.87 ± 1.99^e^	56.05 ± 0.50^a^	15.47 ± 0.57^a^	2.83 ± 0.09^a^
Chemical	13.26 ± 1.97^c^	57.89 ± 0.53^b^	16.31 ± 1.25^b^	2.98 ± 0.18^ab^
C-PF	16.73 ± 1.82^d^	57.87 ± 0.43^b^	16.79 ± 0.78^b^	2.93 ± 0.15^ab^
Bio + 50% Chemical	11.42 ± 1.55^b^	63.42 ± 0.62^c^	18.15 ± 0.56^c^	3.02 ± 0.11^ab^
MSSRFD41	8.39 ± 1.63^a^	64.68 ± 0.77^d^	19.05 ± 0.98^d^	3.23 ± 0.12^b^

*Each value represents the means ± SD (n = 20) and within a column different letters were assigned when differences were significant according to the HSD Tukey test (P < 0.05)*.

### Preparation of liquid-based formulation of MSSRFD41

**Figure 4:**
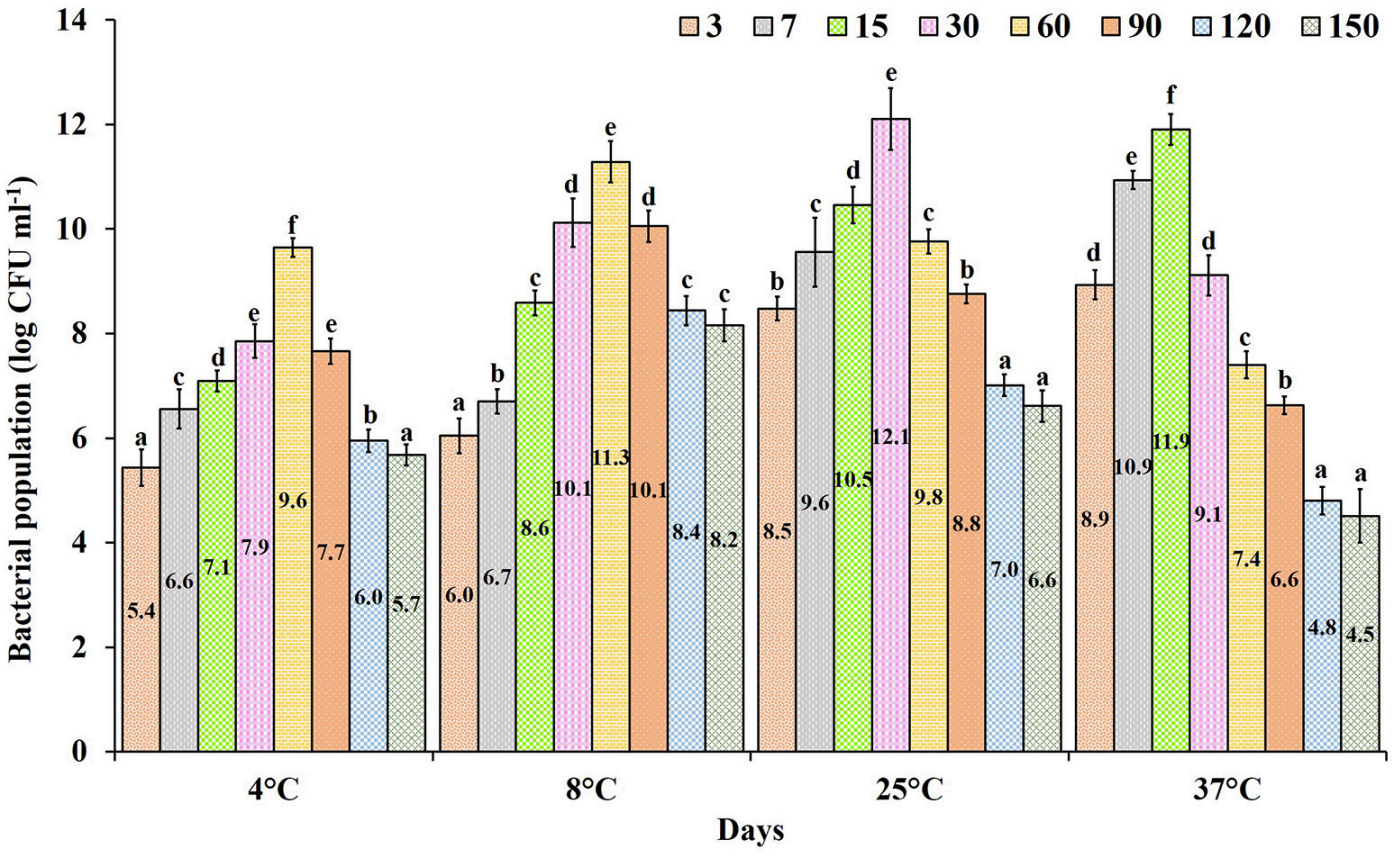
Shelf life of *Pseudomonas* sp. MSSRFD41 in liquid formulated product of different ages incubated at various temperatures. Each value is the mean± *SD* of 3 replicates. Within a column, different letters were assigned when significant according to the HSD Tukey test (*P* < 0.05).

The formulated product incubated at different temperatures showed a gradual increase in population of from 4.5 to 12.1 log CFU ml^−1^. The shelf life of the formulated product incubated at 25°C showed the highest average population of 9.12 log CFU ml^−1^ compared to other tested temperatures, followed by 8°C with an average of 8.67 log CFU ml^−1^ with the highest survival rate at 150 days of incubation (8.2 log CFU ml^−1^). At 37°C the formulated product had an average population level of 8.01 log CFU ml^−1^, which showed higher CFU initially (8.9 log CFU ml^−1^), but a reduction in the population level that occurred from the 30th day of incubation (4.5 log CFU ml^−1^). In products stored at 4 and 8 °C the population level gradually increased (5.4 log CFU ml^−1^) and declined at by the 90th day of incubation (Figure 4). Overall, the product incubated at 8 and 25°C temperature maintained the recommended dosage level till 150th days of incubation without any contamination.

## Discussion

Control of blast disease employing biological agents can enhance agricultural productivity in crops such as wheat, rice, millet, and barley, and is reported to protect yield loss (Kumar and Kumar, 2011; Prajapati et al., 2013; Cruz and Valent, 2017). Bioinoculants are sustainable natural resources with a wide range of disease control strategies and multiple beneficial plant growth promoting traits (Negi et al., 2015; Santhanam et al., 2015; Souza et al., 2015; Cruz and Valent, 2017; Deketelaere et al., 2017; GutiéRrez-GarcíA et al., 2017). Many studies have reported pseudomonads as bioinoculants with the potential to manage phytopathogens and promote the growth of crops cultivated under different agro-climatic conditions (Yin et al., 2013; Selvaraj et al., 2014; Wang et al., 2015). In this study, we have demonstrated the potential of the indigenous rhizospheric *Pseudomonas* sp. strain MSSRFD41 to control blast disease and promote the growth of finger millet.

Strain MSSRFD41 significantly suppressed mycelial growth of *P. grisea* in dual plate culture assays, consistent with antifungal action through the production of secondary metabolites, antibiotics, volatiles and hydrolytic enzymes (Sekar and Prabavathy, 2014). Pseudomonads are known to produce a wide range of metabolites including antibiotics (2,4-DAPG, HCN, PLT, and PCA) and enzymes that exhibit antagonistic activity against phytopathogens (Jegan, 2015; Müller et al., 2016; Vacheron et al., 2017; Yan et al., 2017). A GC/MS profile of MSSRFD41 crude extract showed the presence of reported potential antimicrobial metabolites including pyrrolo [1,2-a] pyrazine-1,4-dione, hexahydro-3-(2-methylpropyl) a major antimicrobial metabolite (Melo et al., 2014), H-pyran, 2-(7-heptadecynyloxy)tetrahydro (Devi and Muthu, 2014); piperidine-3-carboxylic acid (Santiago et al., 2016); tert-hexadecanethiol (Giri et al., 2017); 1-heptatriacotanol (Kalaiarasan et al., 2011); octadecadienoic acid (Ganesh and Mohankumar, 2017); pyrazine and 2,4-DAPG derivatives (GutiéRrez-GarcíA et al., 2017). Production of these groups of potential antifungal secondary metabolites correlates to the efficacy of MSSRFD41 in the inhibition of *P. grisea*. In addition, numerous studies have correlated the antagonistic activity of pseudomonads with production of cell wall lytic enzymes such as chitinase and protease, which are reported to suppress the growth of fungal phytopathogens by degrading the cell wall (Radjacommare et al., 2004; Sekar and Prabavathy, 2014; Negi et al., 2015; Giri et al., 2017).

In this study, ESEM analysis demonstrated that the hyphae of *P. grisea* exposed to soluble substances produced by strain MSSRFD41 were damaged, forming clumps and exuding cellular material outside the cell wall. These images are consistent with the impact of metabolites, volatiles and hydrolytic enzymes from MSSRFD41. Similarly, Li et al. (2014) observed that the natural product citral caused damage to hyphal cell walls and membrane structures of *P. grisea*. The cell walls of *P. grisea* are strongly influenced by hydrophilic and aldehyde groups which damage or rupture cell walls and membranes (Xiong et al., 2013). A few studies also have shown that 2,4-DAPG plays an important role in the control of phytopathogens by damaging hyphal tips, causing vacuolization and cell content disintegration, and disrupting multiple basic cellular pathways including membrane function, reactive oxygen regulation, and cell homeostasis (de Souza et al., 2003; Kwak et al., 2011). Strain MSSRFD41 secretes hydrolytic enzymes including cellulase, chitinase, lipase, protease, and esterase, all of which are capable of causing the damage and lysis of fungal cell walls leading to death (Han et al., 2006; Ariffin et al., 2008; Arunachalam Palaniyandi et al., 2013; Sarma et al., 2014; Negi et al., 2015). Strain MSSRFD41 exhibits multiple modes of action against *P. grisea*, all of which may be a key traits contributing to its efficacy and consistency of performance.

Bioinoculants can enhance the rate of seed germination, plant biomass and vigor index, protect from disease, and exhibit synergistic interactions with the seed and soil microfloral community through direct and indirect traits (Patten and Glick, 2002; Ariffin et al., 2008; Wang et al., 2015; Vacheron et al., 2017). Multiple growth promoting factors have been reported to be responsible for significant responses other than biocontrol in seeds treated with bioinoculants (Raudales et al., 2009; Manikandan et al., 2010; Negi et al., 2015). Finger millet seeds primed with MSSRFD41 were significantly enhanced in germination, seedling emergence, and biomass than control seeds. The ability to produce amylase plays a role in enhancing the rate of seed germination and early growth of millet seeds. It hydrolyzes endosperm starch into sugars, which acts as a source for the growth of roots and shoots in the germinating seedling (Duarah et al., 2011). MSSRFD41 produces the phytohormone IAA, a product which is widely produced by plant-associated bacteria, especially bioinoculants (Patten and Glick, 2002; Zahid et al., 2015). IAA plays significant role in plant signaling pathways to coordinate the physiological and morphological responses. IAA-mediated induction of primary and adventitious root development in young seedlings will augment their establishment in soil and facilitate the uptake of nutrients and water (Patten and Glick, 2002; Verma et al., 2013; Zahid et al., 2015). In addition, IAA plays an important role in the regulation of plant abiotic stresses induced by ethylene. Enhancing the availability of plant-utilizable forms of phosphate, iron, potassium and zinc via solubilisation and mobilization by bioinoculants directly increases nutrient availability in soil, thus increasing soil fertility, plant growth, crop productivity and also nutrient content of the grain (Gupta et al., 2015; Souza et al., 2015; Gopalakrishnan et al., 2016; Jegan et al., 2016; Kamran et al., 2017). The direct and indirect mechanisms of plant growth promotion mediated by strain MSSRFD41 reflect its ability to increase the nutrient content of the seed, soil, and plant biomass.

Currently, seed biopriming is reported as an efficient method of bioinoculant application to protect from seed and soil-borne phytopathogens, increase the speed and uniformity of germination, and plant growth and yield (Patten and Glick, 2002; Raudales et al., 2009; Mahmood et al., 2016). Gopalakrishnan et al. (2016) reported the efficacy of bioinoculants in growth promotion of cereals and legumes with biofortification of mineral nutrients in the grains. In this study, seed treatment with strain MSSRFD41 significantly enhanced biometric observations other than blast control *in vivo*. Pseudomonads are reported widely for their ability to enhance the availability and uptake of plant macro and micronutrients by either mobilization or solubilization from complex resources (Raudales et al., 2009; Goljanian-Tabrizi et al., 2016; Mahmood et al., 2016; Vacheron et al., 2017).

The soil microbiome is involved in the establishment of a stable ecosystem through synergistic interactions of compatible microbes. Rhizobiome communities are often referred to as the plant's second genome (Berendsen et al., 2012). Bioinoculants should be compatible with rhizobiome without adversely impacting the existing microbial community and should significantly improve crop yield, plant growth, and soil fertility (Pandey et al., 2012; Singh et al., 2013; Kröber et al., 2014). Several studies have attempted to determine the compatibility among co-bioinoculants for the development of beneficial consortia, but these studies did not explore the compatibility of bioinoculants against a wide range of rhizobacteria (Pandey et al., 2012; Singh et al., 2013; Zhang et al., 2016; Vacheron et al., 2017). In this study, our cross-streaking assay revealed the compatibility of MSSRFD41 with different native and other rhizobacterial groups without any detectable growth inhibition at the intersection of two colonies. Prior to the application of bioinoculants in the field, the cross streaking assay can strongly suggest that the introduced isolate will be compatible with the indigenous rhizobiome and soil microbiome.

Potential bioinoculants are determined by their efficacy in crop protection, growth promotion, survival in the rhizosphere, and compatibility with the native microbiome (Selvaraj et al., 2014; Gupta et al., 2015; Mahmood et al., 2016; Zhang et al., 2016; Deketelaere et al., 2017). The MSSRFD41 treated finger millet rhizosphere holds higher CFU g^−1^ than control samples but without any significant difference of CFU g^−1^ in both KBA+SLT and KB+CN media. These selective media are reported to use for the selection of pseudomonads from different soil samples and it recover significantly higher number of pseudomonas CFU g^−1^ than normal heterotrophic media (Goto and Enomoto, 1970; Gould et al., 1985; Picard et al., 2000; Thomas and Sekhar, 2016). Difference in colonies number between the treatment and control samples and BOX-PCR analysis may represent survival of MSSRFD41 in the rhizosphere soil of the treated samples. Thomas and Sekhar (2016) used a similar approach with selective media to detect the survival of endophytic *Pseudomonas aeruginosa* and its impact in soil from the banana rhizosphere. Recently, Sharma et al. (2017) described bioinoculants that survived in the rhizosphere, were compatible with other groups of rhizobacteria, and enhanced pigeon pea yield. Yin et al. (2013) also reported no detectable alteration of the native cucumber rhizobacterial community treated with *Pseudomonas fluorescens* 2P24 and CPF10 bioinoculants by using a culturable approach, T-RFLP, and DGGE. Bazhanov et al. (2017) used BOX-PCR approach to determine the colonization of inoculated atrazine-degrading strain *Arthrobacter ureafaciens* DnL1-1 from the roots of treated plants. Along with the results of culturable analysis, the RAPD fingerprinting pattern in our study also indicated the survival of strain MSSRFD41 in treated rhizosphere samples by forming a monomorphic fingerprinting pattern with MSSRFD41 reference DNA, but absent in control samples. In addition, this fingerprinting analysis revealed the existence of similar rhizobacterial communities in both the treated and control rhizosphere soils without a detectable change in the existing bacterial community. Phylogenetic analysis of 16S rRNA also evidenced the existence of MSSRFD41 in the rhizosphere of treated plants. These results support the idea that strain MSSRFD41 survived in the rhizosphere by establishing a symbiotic relationship with the native rhizobiome and soil microbial communities. Similar to our results, Gupta et al. (2015) reported that a consortium of bioinoculants including *Bacillus megaterium, Pseudomonas fluorescens*, and *Trichoderma harzianum* influenced the yield of pigeon pea without a negative impact on the native rhizospheric microbial community. PLFA analysis also indicated that the total biomass, including contents associated with Gram-positive and Gram-negative bacteria, actinomycetes, fungi, and eukaryotes, was not significantly decreased by treatment with strain MSSRFD41. In many studies, PLFA biomarkers provide an indicator for assessing living microbial biomass to detect the effects of treatments on soil microbial community composition (Paterson et al., 2007; Buyer and Sasser, 2012; Zhang et al., 2016).

Several studies have reported a gradual reduction of bioinoculant populations and the native rhizobiome community in soil (Schreiter et al., 2014; Thomas and Sekhar, 2016). On the other hand, studies have shown that bioinoculants maintain a more stable soil microbiome, enhance plant growth, and control phytopathogenic infections more effectively than chemical pesticides and mineral fertilizer applications (Chowdhury et al., 2013; Kröber et al., 2014; Souza et al., 2015; Cai et al., 2017). In this study MSSRFD41 established in the rhizosphere and might survive through its communication signaling system as it is reported to produce AHL quorum sensing (QS) molecules (Sekar and Prabavathy, 2014). Several studies reported QS signaling system plays an important role in the establishment, survival, and gene regulation of bacterial community in the rhizosphere region (Boyer and Wisniewski-Dyé, 2009; Hartmann et al., 2014; Ganga et al., 2016).

Our results from cross-streaking, and culturable, RAPD, and PLFA analyses are all consistent with the hypothesis that strain MSSRFD41 survived, is compatible with the rhizobiome, has no detectable effect on the soil microbial community, and enhanced plant growth. Though all the analyses provided preliminary qualitative data on the differences between the control and treated rhizosphere samples, but does not provide quantitative data on the composition of microbial community. Approaches like next-generation sequencing, markers, fluorescent *in situ* hybridization (FISH), and molecular fingerprinting methods will be necessary to strongly prove the survivability and also for further investigation. In particular, marker approaches will play a significant role in determination of the fate of bioinoculants in the rhizosphere. Mosimann et al. (2016) demonstrated the colonization rate and quantitatively assessed the persistence of a bioinoculants in the maize root and rhizoplane through TaqMan-based quantitative PCR.

Studies to evaluate the impact of bioinoculants for the control of blast disease in the finger millet have shown disease suppression in the range of 16–54% (Radjacommare et al., 2004; Kumar and Kumar, 2011; Waghunde et al., 2013; Negi et al., 2015). A field experiment in this study revealed that inoculation with the native finger millet strain MSSRFD41 resulted in 8.39% disease incidence, which was significantly better than other treatments including a chemical fungicide. Several studies reported that foliar application of pseudomonads had an ability to act at the site of pathogenic infestation by damaging the fungal cell wall and retarded growth through a network of interconnecting signal leading to accumulation of defense-related enzymes and proteins via, induced systemic resistance (ISR) and systemic acquired resistance (SAR) systems (Bahadur et al., 2007; De Vleesschauwer et al., 2008; Spence et al., 2014; Negi et al., 2015; Fatima and Anjum, 2017; Yasmin et al., 2017). In addition, studies also showed that extracellular metabolites such as 2,4-DAPG, HCN, cyclic lipopeptides, defense proteins, peroxidase (POD), catalase (CAT), phenylalanine ammonia-lyase (PAL) and polyphenol oxidase (PPO) produced by bioinoculants have been found to be responsible for antifungal activity during aerial infestation (Waghunde et al., 2013; Yin et al., 2013; Negi et al., 2015; Wang et al., 2015; Mahmood et al., 2016; Müller et al., 2016).

The combination of bioinoculants and chemical treatment to minimize the application of fungicide was less effective in controlling blast disease than treatment with strain MSSRFD41. In addition, strain MSSRFD41 was more effective against blast and in promoting growth than were chemical, C-PF and control treatments. Studies have observed similar impacts in different crops with a combination of multiple bioinoculants and chemical fungicides (Senthil et al., 2012; Waghunde et al., 2013; Magar et al., 2015).

Though bioinoculants protect plants from pathogens and promote growth significantly under lab conditions, efficacy often is less under greenhouse and field conditions (Lugtenberg and Kamilova, 2009). The use of a bioinoculant indigenous to the host plant is promising because of its reported high success rate in the establishment, significant growth promotion, and disease control (Verma et al., 2013; Santhanam et al., 2015; Zahid et al., 2015). Strain MSSRFD41 improved plant shoot length of 19.58% in paper towel assay; 13.60% and 14.29% in pot; and field conditions; root length of 11.65% in paper towel assay; 25.62% in pot; 21.12% under field conditions as compared to control plants.

The efficacy of MSSRFD41 was achieved by establishing in the rhizosphere through seed priming and root dipping which provided protection to the finger millet against blast disease in the initial growth stage and foliar spray at later growth stage through induced systematic resistance. In our earlier work we reported MSSRFD41 as a novel 2,4-DAPG producing genotype G with the production of 40 μg ml^−1^ (Sekar and Prabavathy, 2014). There are strong evidence that 2,4-DAPG and elicitors plays an vital role in the control of phytopathogenic infections by lysis the fungal hyphae and induction of plant immune system under greenhouse and field conditions (Weller et al., 2012; Negi et al., 2015; Wang et al., 2015; Ganga et al., 2016; Vacheron et al., 2017; Yan et al., 2017).

The consistency and efficacy of strain MSSRFD41 in crop protection and growth promotion is critical if it is to be developed as a bioinoculants. Such bioinoculants must exhibit a prolonged shelf life in a viable form. Several reports have stated that liquid formulation is more efficient than other types of formulation such as talc, encapsulation, and jelly, because of its easy application, higher efficacy, potential shelf life, low level of contamination, and higher field performance (Hedge, 2002; Selvaraj et al., 2014). Most research on shelf life has focused on the stability of liquid formulations for different lengths of time at constant temperature (Manikandan et al., 2010; Selvaraj et al., 2014; Goljanian-Tabrizi et al., 2016). In this study, the impact of both the duration of storage and the temperature of a formulated preparation of strain MSSRFD41 revealed significant changes in the population over time. At 25°C an average of 10^7^ CFU ml^−1^ was maintained over 150 days, strongly suggesting the suitability of the liquid formulation for storage at room temperature. Manikandan et al. (2010) also reported that the shelf life of *P. fluorescens* in a glycerol-amended liquid formulation was maintained for up to 6 months at 25°C.

## Conclusion

The present study systematically investigated the potential of an indigenous rhizobacterium of finger millet, *Pseudomonas* sp. MSSRFD41, for control of blast disease and growth promotion in finger millet. Our results suggest MSSRFD41 exhibited multiple beneficial traits to the host plants and results of compatibility and survival assessment revealed that MSSRFD41 could be a substitute and sustainable resource to reduce the usage of chemical fungicides for control of blast disease on finger millet. Further, the efficacy of the formulated product remains to be determined by conducting multi-location trials at different seasons. In addition, a biosafety assessment is needed before the formulated product can be developed commercially.

## Author contributions

JS designed and performed experiments, field trial, data collection, statistical analysis, and manuscript write-up. KR and PD were involved in sample collection, contributed to *in vitro* experiments, analysis tools, and manuscript correction. PR supervised and supported the study, and revised and approved the final version of the manuscript to be published. All authors proofread and reviewed the manuscript.

### Conflict of interest statement

The authors declare that the research was conducted in the absence of any commercial or financial relationships that could be construed as a potential conflict of interest.

## Abbreviations

2, 4-DAPG: 2,4-diacetylphloroglucinol
PGPR: plant growth-promoting rhizobacteria
Biopriming
IAA: Indole-3-acetic acid
*P. grisea*
Blast
ESEM: Environmental scanning electron microscopy.
