# Supplementary figures and images for: Potential of Finger Millet Indigenous Rhizobacterium Pseudomonas sp. MSSRFD41 in Blast Disease Management—Growth Promotion and Compatibility With the Resident Rhizomicrobiome
Source: Front Microbiol. 2018 May 23;9:1029. doi: 10.3389/fmicb.2018.01029 (PMC5974220; doi:10.3389/fmicb.2018.01029)

Fig. S1. Randomized block design (RBD) of field trial.

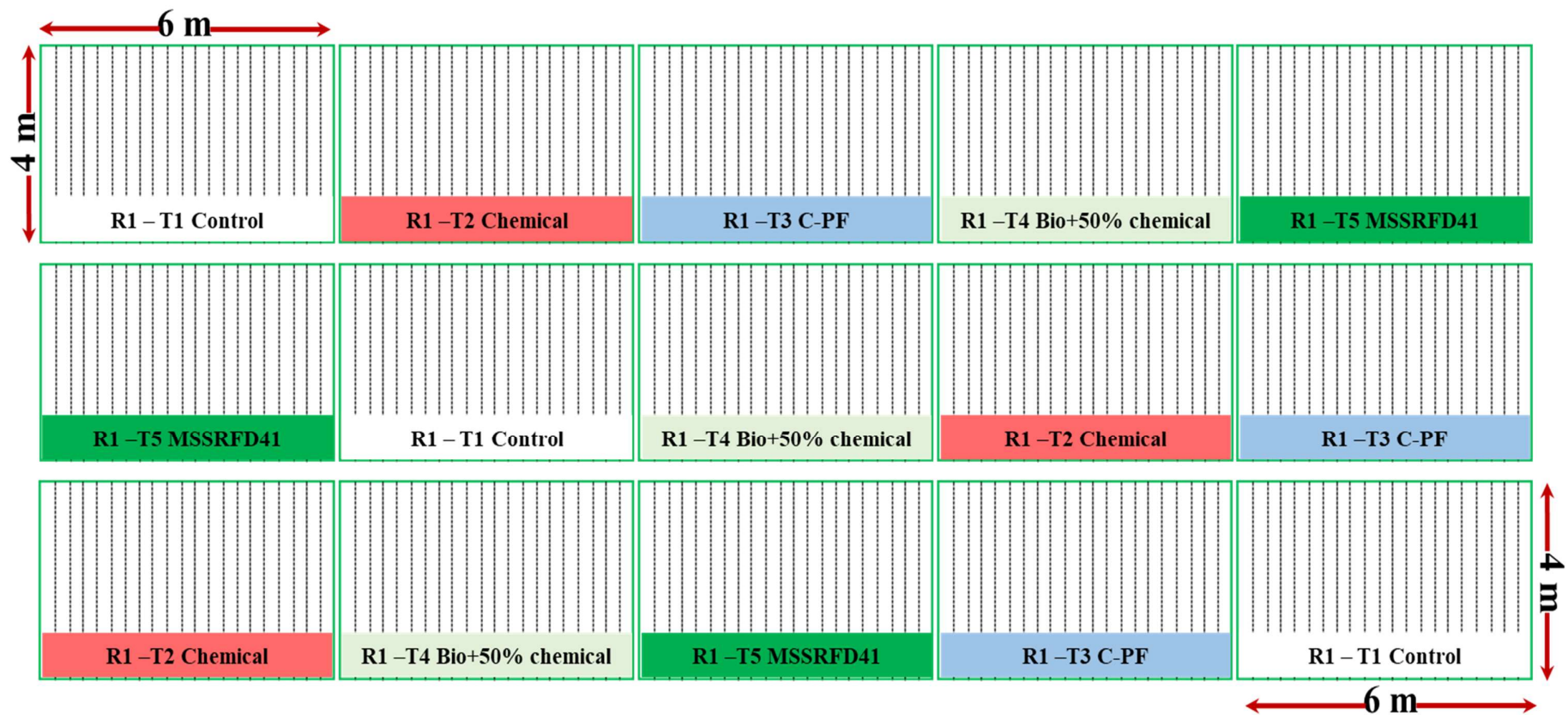

Supplement: Supplementary file 1 [file Image_1.PDF]

**Fig. S5. Impact of MSSRFD41 treatment in finger millet growth in pot at 60<sup>th</sup> day.**

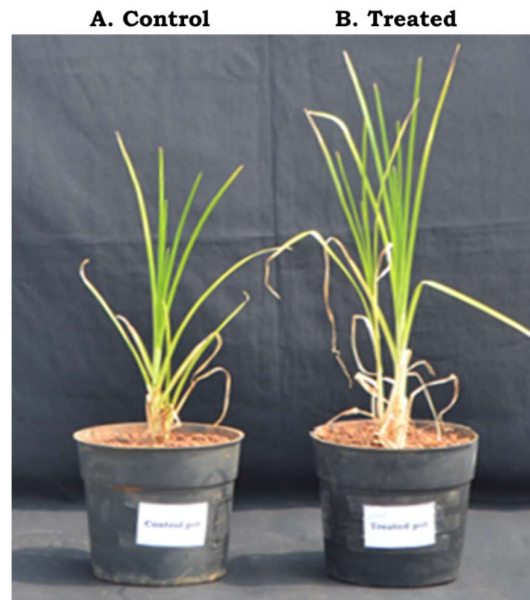

Supplement: Supplementary file 5 [file Image_5.PDF]
