# Supplementary material for: Potential of Finger Millet Indigenous Rhizobacterium Pseudomonas sp. MSSRFD41 in Blast Disease Management—Growth Promotion and Compatibility With the Resident Rhizomicrobiome
Source: Front Microbiol. 2018 May 23;9:1029. doi: 10.3389/fmicb.2018.01029 (PMC5974220; doi:10.3389/fmicb.2018.01029)
Supplement: Supplementary file 2 [file Image_2.PDF]

Fig. S2. Compatibility of MSSRFD41 against different rhizobacterial isolates.

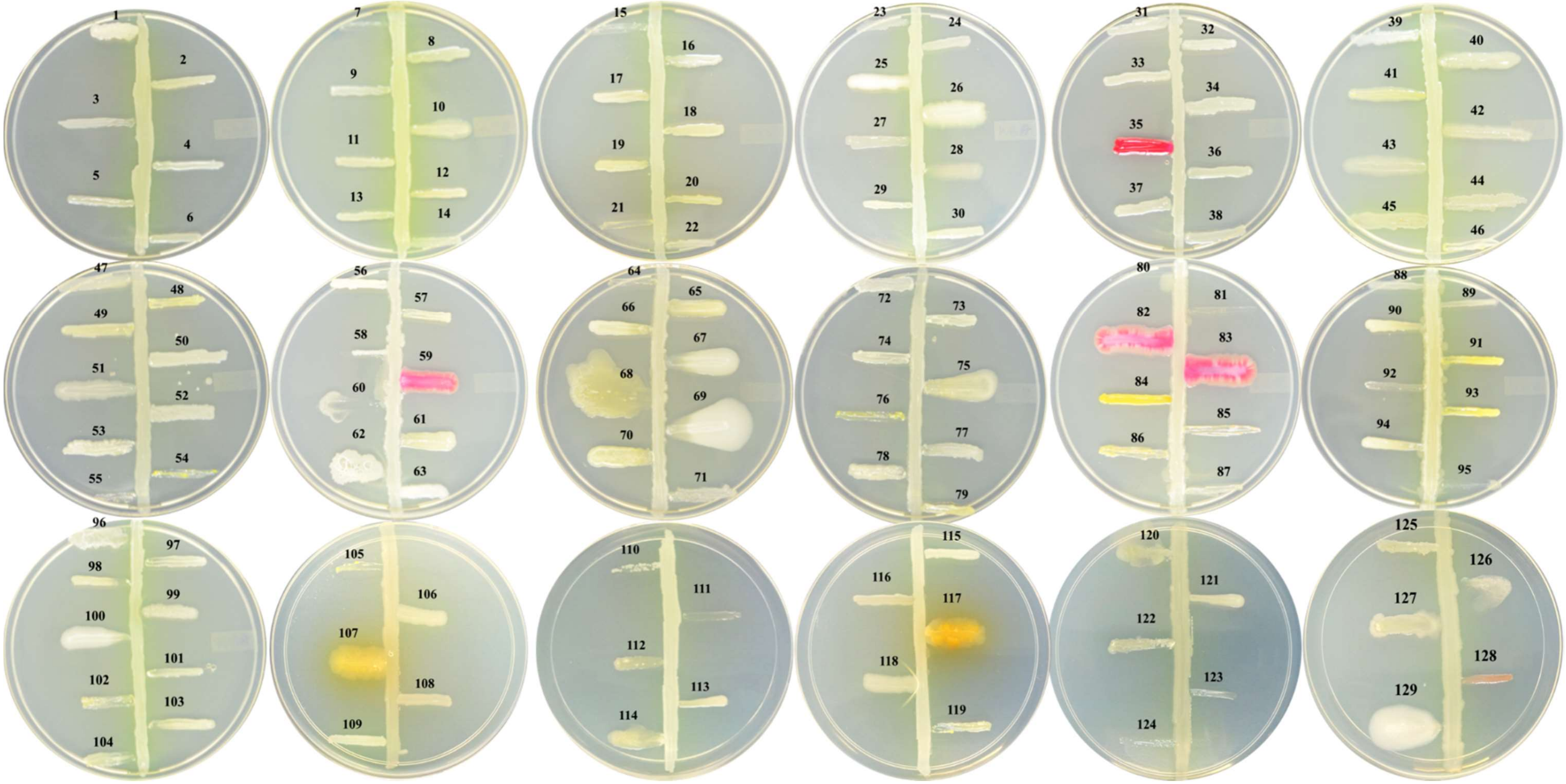

Cross-streak between MSSRFD41 and other rhizobacterial isolates. MSSRFD41 is streaked in middle and adjacent strains are isolated from the rhizosphere of finger millet, wheat, paddy, groundnut, pepper, *Poteresia*, pigeon pea, *Thypa*, *Canna*, different soil and water sources.
