# Supplementary material for: Potential of Finger Millet Indigenous Rhizobacterium Pseudomonas sp. MSSRFD41 in Blast Disease Management—Growth Promotion and Compatibility With the Resident Rhizomicrobiome
Source: Front Microbiol. 2018 May 23;9:1029. doi: 10.3389/fmicb.2018.01029 (PMC5974220; doi:10.3389/fmicb.2018.01029)
Supplement: Supplementary file 3 [file Image_3.PDF]

Fig. S3. Impact of MSSRFD41 biopriming in root and shoot development of finger millet seeds at 8th day.

A. Control **B. Treated**

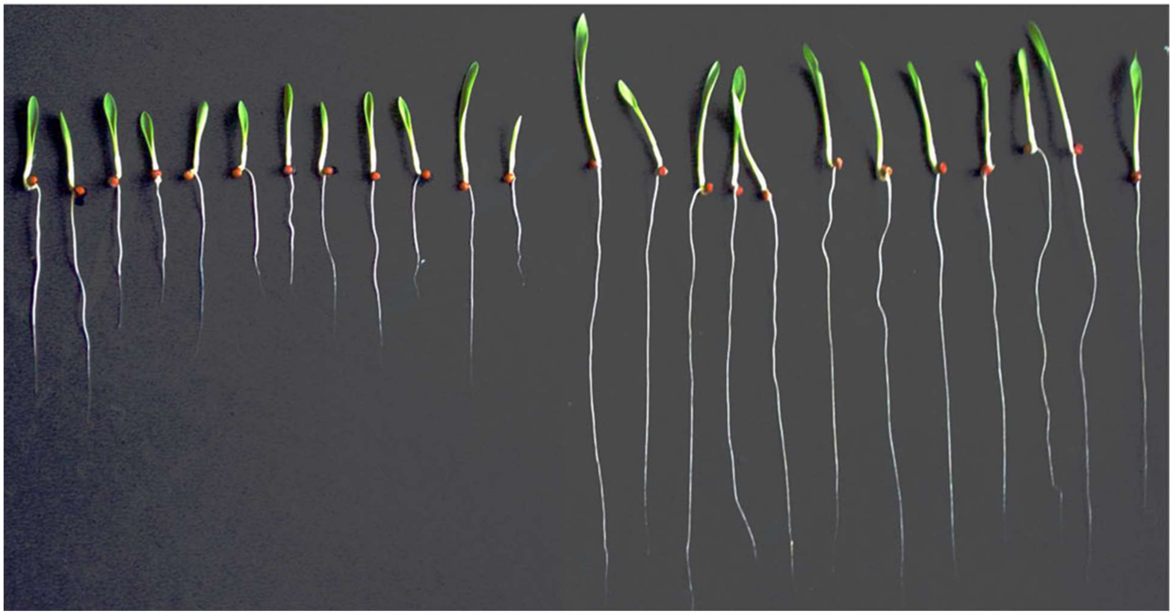

A. Control millet seeds are treated with sterile 0.03 M $MgSO_4$, B. MSSRFD41 primed millet seeds which showed enhancement of root and shoot development than control seeds.
