# Supplementary material for: Potential of Finger Millet Indigenous Rhizobacterium Pseudomonas sp. MSSRFD41 in Blast Disease Management—Growth Promotion and Compatibility With the Resident Rhizomicrobiome
Source: Front Microbiol. 2018 May 23;9:1029. doi: 10.3389/fmicb.2018.01029 (PMC5974220; doi:10.3389/fmicb.2018.01029)
Supplement: Supplementary file 4 [file Image_4.PDF]

**Fig. S4. Phylogenetic tree based on 16S rRNA gene sequences showing the evolutionary relationship of rhizobacterial and MSSRFD41 isolates.**

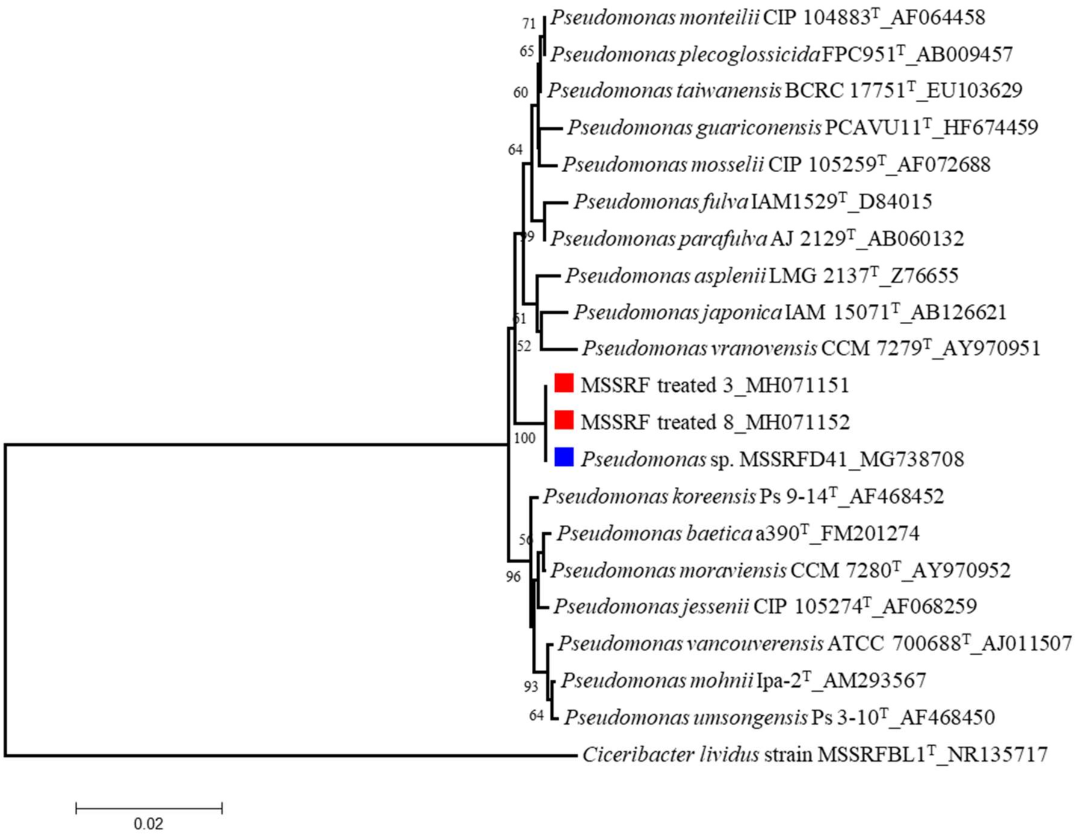


**Rhizobacteria isolated from MSSRFD41 treated pots; MSSRFD41 reference**

The evolutionary history was inferred using the Neighbor-Joining and Maximum Composite Likelihood method with branch length of 0.12. The percentages shown at each branching point represent bootstrap values derived from 1,000 replications and values of more than 50% are indicated.
