# Supplementary material for: Potential of Finger Millet Indigenous Rhizobacterium Pseudomonas sp. MSSRFD41 in Blast Disease Management—Growth Promotion and Compatibility With the Resident Rhizomicrobiome
Source: Front Microbiol. 2018 May 23;9:1029. doi: 10.3389/fmicb.2018.01029 (PMC5974220; doi:10.3389/fmicb.2018.01029)
Supplement: Supplementary file 6 [file Table_1.PDF]

Supplementary table 1. List of bacterial strains from different sources obtained from the MSSRF culture center for the cross streaking assay with MSSRFD41.

S. No	Strain No	Taxonomy	Source	Accession ID
1	MSSRFPD15	*Acinetobacter* sp.	Canna rhizosphere	KX901870
2	MSSRFPD1	*Pseudomonas guariconensis*	Canna rhizosphere	KX901856
3	MSSRFPD23	*Alcaligenes* sp.	Bulk soil	KX901878
4	MSSRFPD20	*Alcaligenes* sp.	Bulk soil	KX901875
5	MSSRFPH124	*Glutamicibacter nicotianae*	Typha rhizosphere	-
6	MSSRFPH203	Unidentified	Bulk soil	-
7	MSSRFPD32	*Lysinibacillus mangiferihumi*	Wastewater	KX901887
8	MSSRFPD25	*Ralstonia pickettii*	Canna rhizosphere	KX901880
9	MSSRFPD22	*Alcaligenes* sp.	Bulk soil	KX901877
10	MSSRFPD7	*Pseudomonas jinjuensis*	Canna rhizosphere	KX901862
11	MSSRFPD11	*Pseudomonas jinjuensis*	Canna rhizosphere	KX901866
12	MSSRFPD13	*Stenotrophomonas nitritireducens*	Bulk soil	KX901868
13	MSSRFPBP1	Unidentified	Canna rhizosphere	-
14	MSSRFPD25	*Ralstonia pickettii*	Canna rhizosphere	KX901880
15	MSSRFPD20	*Alcaligenes* sp.	Bulk soil	KX901875
16	MSSRFPD33	*Rhodococcus* sp.	Typha rhizosphere	KY849349
17	MSSRFPD35	*Glutamicibacter nicotianae*	Canna rhizosphere	KY849351
18	MSSRFPH126	*Glutamicibacter nicotianae*	Canna rhizosphere	KY849352
19	MSSRFPH143	*Glutamicibacter nicotianae*	Canna rhizosphere	KX901884
20	MSSRFPD17	*Acinetobacter indicus*	Bulk soil	KX901872
21	MSSRFPD24	*Cupriavidus oxalaticus*	Canna rhizosphere	KX901879
22	MSSRFPD32	*Lysinibacillus mangiferihumi*	Wastewater	KX901887
23	MSSRFPD3	*Pseudomonas knackmussii*	Canna rhizosphere	KX901858
24	MSSRFPD9	*Pseudomonas jinjuensis*	Canna rhizosphere	KX901864
25	MSSRFPD4	*Pseudomonas monteilii*	Canna rhizosphere	KX901859
26	MSSRFPD2	*Pseudomonas plecoglossicida*	Canna rhizosphere	KX901857
27	MSSRFPD3	*Pseudomonas knackmussii*	Canna rhizosphere	KX901858
28	MSSRFPD34	*Pseudomonas knackmussii*	Typha rhizosphere	KY849350
29	MSSRFPD35	*Glutamicibacter nicotianae*	Canna rhizosphere	KY849351
30	MSSRFPH126	Unidentified	Typha rhizosphere	-
31	MSSRFHR2	Unidentified	Finger millet rhizosphere	-
32	MSSRFHL2	Unidentified	Finger millet rhizosphere	-
33	MSSRFHL3	Unidentified	Finger millet rhizosphere	-
34	MSSRFHL1	Unidentified	Finger millet rhizosphere	-
35	MSSRFHR1	*Serratia* sp.	Finger millet rhizosphere	-

S. No	Strain No	Taxonomy	Source	Accession ID
36	MSSRFHR3	Unidentified	Finger millet rhizosphere	-
37	MSSRFHL4	Unidentified	Finger millet rhizosphere	-
38	MSSRFHR4	Unidentified	Finger millet rhizosphere	-
39	MSSRFV343	*Bacillus megaterium*	Finger millet rhizosphere	JF784650
40	BCRC17751T	*Pseudomonas taiwanensis*	Soil	EU103629
41	NBRC 103040^T	*Pseudomonas japonica*	Sludge	BBIR01000146
42	NBRC 16636^T	*Pseudomonas parafulva*	Paddy rhizosphere	BBIU01000051
43	ATCC 23835^T	*Pseudomonas asplenii*	Soil	LT629777
44	NBRC 14164^T	*Pseudomonas putida*	Water	AP013070
45	ATCC 700688^T	*Pseudomonas vancouverensis*	Forest soil	AJ011507
46	MSSRFD865	*Pseudomonas* sp.	Finger millet rhizosphere	HQ455013
47	DSM 18327^T	*Pseudomonas mohnii*	Water	FNRV01000001
48	LMG 27394^T	*Pseudomonas guariconensis*	Cowpea rhizosphere	FMYX01000029
49	MSSRFJ3	Unidentified	Brinjal rhizosphere	-
50	MSSRFJ41B	Unidentified	Brinjal rhizosphere	-
51	MSSRFYU7	Unidentified	Brinjal rhizosphere	-
52	MSSRFYU8	Unidentified	Brinjal rhizosphere	-
53	MSSRFYU9	Unidentified	Brinjal rhizosphere	-
54	MSSRFYU10	Unidentified	Brinjal rhizosphere	-
55	MSSRFYU11	Unidentified	Brinjal rhizosphere	-
56	MSSRFSD1	*Pseudomonas indica*	Finger millet rhizosphere	-
57	MSSRF1B8A	*Rhizobium* sp.	Black bean rhizosphere	-
58	MSSRF2A33	*Rhizobium* sp.	Red bean nodules	-
59	MSSRF1B39	*Methylobacterium populi*	Black bean nodules	-
60	MSSRFH104	*Bacillus asahii*	Wheat rhizosphere	HF952539
61	MSSRFD182	*Bacillus cereus*	Finger millet rhizosphere	HQ454986
62	MSSRFD148	*Bacillus endophyticus*	Finger millet rhizosphere	JF784642
63	MSSRFD491	*Bacillus firmus*	Finger millet rhizosphere	JF784646
64	MSSRFD182	*Bacillus cereus*	Finger millet rhizosphere	JF784645
65	MSSRFV287	*Bacillus pumilus*	Finger millet rhizosphere	JF784649
66	MSSRFV343	*Bacillus megaterium*	Finger millet rhizosphere	JF784650
67	MSSRFD263	*Bacillus pumilus*	Finger millet rhizosphere	JF784644
68	MSSRFD538	*Bacillus cereus*	Finger millet rhizosphere	JF784647
69	MSSRFNM2	*Bacillus* sp.	Avicennia rhizosphere	-
70	MSSRFNM3	*Bacillus* sp.	Avicennia rhizosphere	-
71	MSSRFD256	*Pseudomonas alcaligenes*	Finger millet rhizosphere	-
72	MSSRF2A2	Unidentified	Red bean nodules	-
73	MSSRF1A23	Unidentified	Black bean nodules	-

S. No	Strain No	Taxonomy	Source	Accession ID
74	MSSRF1A48A	Unidentified	Black bean nodules	-
75	MSSRF2A59B	Unidentified	Red bean nodules	-
76	MSSRFT6	Unidentified	Tomato rhizosphere	-
77	MSSRFT15	*Brevibacillus* sp.	Tomato rhizosphere	-
78	MSSRFT100	Unidentified	Tomato rhizosphere	-
79	MSSRFMSD16	Unidentified	Tomato rhizosphere	-
80	MSSRF2A59	*Rhizobium etli*	Red bean nodules	MG597198
81	MSSRF2A30A	*Rhizobium etli*	Red bean nodules	MG597199
82	MSSRFG18	Unidentified	Avicennia rhizosphere	-
83	MSSRFG28	Unidentified	Avicennia rhizosphere	-
84	MSSRFG58	Unidentified	Avicennia rhizosphere	-
85	MSSRFDE368	Unidentified	Pigeon pea rhizosphere	-
86	MSSRFMET1	*Methylobacterium* sp.	Tomato rhizosphere	-
87	MSSRFNM1	Unidentified	Avicennia rhizosphere	-
88	MSSRFDE293	Unidentified	Pigeon pea rhizosphere	-
89	MSSRFDE304	Unidentified	Pigeon pea rhizosphere	-
90	MSSRFDE2	Unidentified	Pigeon pea rhizosphere	-
91	MSSRFDE5	Unidentified	Pigeon pea rhizosphere	-
92	MSSRFDE6	Unidentified	Pigeon pea rhizosphere	-
93	MSSRFDE7	Unidentified	Pigeon pea rhizosphere	-
94	MSSRFDE9	Unidentified	Pigeon pea rhizosphere	-
95	MSSRFDE10	Unidentified	Pigeon pea rhizosphere	-
96	MSSRF2A3	*Rhizobium* sp.	Red bean nodules	-
97	MSSRF1B48	*Rhizobium* sp.	Black bean rhizosphere	-
98	MSSRF2A9	*Rhizobium* sp.	Red bean nodules	-
99	MSSRF2A4	*Rhizobium* sp.	Red bean nodules	-
100	MSSRF1A66	*Paenarthrobacter ureafaciens*	Red bean nodules	MG597193
101	MSSRF1B48	*Rhizobium* sp.	Black bean rhizosphere	-
102	MSSRF2A16	*Rhizobium* sp.	Red bean nodules	-
103	MSSRF2A55	*Rhizobium* sp.	Red bean nodules	-
104	MSSRF1B8B	*Rhizobium* sp.	Red bean rhizosphere	-
105	MSSRFPT1	*Pseudomonas* sp.	Bulk soil	-
106	MSSRFH30	*Pseudomonas* sp.	Bulk soil	-
107	MSSRFH6	*Pseudomonas chlororaphis*	Wheat rhizosphere	HF952533
108	CHA0	*Pseudomonas protegens*	Tobacco rhizosphere	NR114749
109	MSSRFH151	*Pseudomonas fluorescens*	Wheat rhizosphere	HF952544
110	MSSRFCS99	*Streptomyces* sp.	Rice rhizosphere	-
111	MSSRFQS69	*Aeromonas popoffii*	Rice rhizosphere	KJ877658
112	MSSRFQS73	*Aeromonas dhakensis*	Rice rhizosphere	KJ877660

S. No	Strain No	Taxonomy	Source	Accession ID
113	MSSEFQS79	*Aeromonas veronii*	Rice rhizosphere	KJ877652
114	MSSRFQS77	*Rahnella aquatilis*	Rice rhizosphere	KJ877662
115	MSSRFQS99	*Acinetobacter calcoaceticus*	Wheat rhizosphere	HF952535
116	MSSRFH95	*Pseudomonas extremaustralis*	Wheat rhizosphere	HF952538
117	MSSRFH110	*Pseudomonas brassicacearum*	Wheat rhizosphere	HF952540
118	MSSRFH3	*Pseudomonas brassicacearum*	Wheat rhizosphere	HF952531
119	MSSRFH11	*Pseudomonas chlororaphis*	Wheat rhizosphere	HF952534
120	MSSRFD68	*Pseudomonas* sp.	Finger millet rhizosphere	HF952523
121	MSSRFD114	*Pseudomonas* sp.	Finger millet rhizosphere	HF952529
122	MSSRFD256	*Pseudomonas* sp.	Finger millet rhizosphere	HF952530
123	MSSRFD821	*Pseudomonas* sp.	Finger millet rhizosphere	HQ455010
124	MSSRFD845	*Pseudomonas* sp.	Finger millet rhizosphere	HQ455011
125	MSSRFC759	Unidentified	Tomato rhizosphere	-
126	MSSRFC793	Unidentified	Tomato rhizosphere	-
127	MSSRFC809	Unidentified	Pepper rhizosphere	-
128	MSSRFC875	Unidentified	Pepper rhizosphere	-
129	MSSRFC962	Unidentified	Pepper rhizosphere	-
